# Supplementary material for: Risk Factors Associated with the Development of Hospital-Acquired Infections in Hospitalized Patients with Severe COVID-19
Source: Antibiotics (Basel). 2023 Jun 27;12(7):1108. doi: 10.3390/antibiotics12071108 (PMC10376785; doi:10.3390/antibiotics12071108)
Supplement: Supplementary file 1 [file antibiotics-12-01108-s001.zip › antibiotics-2469094-supplementary.pdf]

Supplementary material

Risk Factors Associated with the Development of Hospital-Acquired Infections in Hospitalized Patients with Severe COVID-19

Fernando Solís-Huerta [1,†], Bernardo Alfonso Martinez-Guerra [2,*,†], Carla Marina Roman-Montes [2], Karla Maria Tamez-Torres [2], Sandra Rajme-Lopez [2], Narciso Ortíz-Conchi [3], Norma Irene López-García [3], Guadalupe Yvonne Villalobos-Zapata [3], Andrea Rangel-Cordero [3], Janet Santiago-Cruz [3], Luis Fernando Xancal-Salvador [3], Steven Méndez-Ramos [3], Eric Ochoa-Hein [4], Arturo Galindo-Fraga [4], Alfredo Ponce-de-Leon [2], Maria Fernanda Gonzalez-Lara [3] and Jose Sifuentes-Osornio [5,*]

[1] Instituto Nacional de Ciencias Médicas y Nutrición Salvador Zubirán, Department of Medicine, Mexico City 14080, Mexico; fernando.solish@incmnsz.mx (F.S.-H)
[2] Instituto Nacional de Ciencias Médicas y Nutrición Salvador Zubirán, Department of Infectious Diseases, Mexico City 14080, Mexico; carla.romanm@incmnsz.mx (C.M.R.-M.); karla.tamezt@incmnsz.mx (K.M.T.-T.); sandra.rajmel@incmnsz@mx (S.R.-L.); luis.ponceg@incmnsz.mx (A.P.-d.-L.)
[3] Instituto Nacional de Ciencias Médicas y Nutrición Salvador Zubirán, Department of Infectious Diseases, Clinical Microbiology Laboratory, Mexico City 14080, Mexico; narciso.ortizc@incmnsz.mx (N.O.-C.); irene.lopezg@incmnsz.mx (N.I.L.-G.); yvonne.villalobosz@incmnsz.mx (G.Y.V.-Z.); andrea.rangelc@incmnsz.mx (A.R.-C.); janet.santiagoc@incmnsz.mx (J.S.-C.); fernando.xancals@incmnsz.mx (L.F.X.-S.); steven.mendezr@incmnsz.mx (S.M.-R.); fernanda.gonzalezl@incmnsz.mx (M.F.G.-L.)
[4] Instituto Nacional de Ciencias Médicas y Nutrición Salvador Zubirán, Hospital Epidemiology Department, Mexico City 14080, Mexico; eric.ochoah@incmnsz.mx (E.O.-H.); arturo.galindof@incmnsz.mx (A.G.-F.)
[5] Instituto Nacional de Ciencias Médicas y Nutrición Salvador Zubirán, General Direction, Mexico City 14080, Mexico
† These authors contributed equally to this work.
* Correspondence: bernardo.martinezg@incmnsz.mx (B.A.M.-G.); jose.sifuenteso@incmnsz.mx (J.S.-O.); Tel.: +52-55-54-87-09-00 (ext. 5860) (B.A.M.-G.); +52-55-54-87-09-00 (ext. 6904) (J.S.-O.)

Table S1. Bacterial isolates

**Infection**	**Microorganism**
Hospital-acquired pneumonia/Ventilator-associated pneumonia<br>250 isolates in 173 episodes	*Enterobacter* complex<br>n=49
	Pseudomonas aeruginosa<br>n=35
	Staphylococcus aureus<br>n=32
	Escherichia coli<br>n=29
	Klebsiella pneumoniae<br>n=26
	Klebsiella oxytoca<br>n=25
	Pseudomonas spp<br>n=8
	Stenotrophomonas maltophilia<br>n=8
	Klebsiella aerogenes<br>n=5
	Serratia marcescens<br>n=5
	Acinetobacter spp<br>n=5
	Streptococcus pneumoniae<br>n=5
	Streptococcus spp<br>n=5
	Klebsiella variicola<br>n=3
	Citrobacter spp<br>n=3
	Haemophilus influenzae<br>n=2
	Proteus spp<br>n=2
	Burkhorderia spp<br>n=2
	Morganella morganii<br>n=1
	Raoutella spp<br>n=1
	Enterococcus spp<br>n=1
Primary bloodstream infections<br>66 isolates in 66 episodes	Coagulase-negative *Staphylococcus*<br>n=26
	Enterococcus spp<br>n=12
	Enterobacter complex<br>n=9
	Streptococcus spp<br>n=5
	Staphylococcus aureus<br>n=3
	Escherichia coli<br>n=3
	Klebsiella oxytoca<br>n=2
	Klebsiella aerogenes

	n=2
	Pseudomonas aeruginosa<br>n=2
	Pseudomonas spp<br>n=1
	Klebsiella pneumoniae<br>n=1
	Stenotrophomonas maltophilia<br>n=1
	Mycobacterium chelonae<br>n=1
Bone, joint and skin and soft tissues infections<br>11 isolates in 4 episodes	*Escherichia coli*<br>n=3
	Enterococcus spp<br>n=3
	Morganella morganii<br>n=2
	Enterobacter complex<br>n=1
	Klebsiella pneumoniae<br>n=1
	Pseudomonas aeruginosa<br>n=1
Abdominal infections<br>3 isolates in 1 episode	*Escherichia coli*<br>n=1
	Enterococcus faecalis<br>n=1
	Pseudomonas aeruginosa<br>n=1
Urinary tract infection<br>2 isolates in 2 episodes	*Escherichia coli*<br>n=2
Clostridioides difficille diarrhea<br>1 episode	NA

Table S2. Fungal isolates

Infection	Microorganism
Probable/proven COVID-19 associated pulmonary aspergillosis<br>28 isolates in 29 episodes	*Aspergillus fumigatus*<br>n=11
	Aspergillus spp<br>n=6
	Aspergillus niger<br>n=5
	Aspergillus flavus<br>n=4
	Aspergillus lentulus<br>n=1
	Aspergillus terreus<br>n=1
	Positive *Galactomannan* antigen<br>n=2
Candidemia<br>23 isolates in 17 episodes	*Candida parapsilosis*<br>n=12
	Candida albicans<br>n=9
	Candida tropicalis<br>n=1
	Candida glabrata<br>n=1
Mucormycosis<br>6 isolates in 3 episodes	*Mucor spp*<br>n=4
	Rhizopus spp<br>n=1
	Conidioboluis<br>n=1

Table S3. Antimicrobial susceptibility

**Gram negative rods**	**Third generation cephalosporin resistant**	**Carbapenem resistant**
Enterobacter complex<br>n=62	62	0
Escherichia coli<br>n=46	23	4
Klebsiella pneumoniae<br>n=31	7	0
Klebsiella oxytoca<br>n=27	1	0
Klebsiella aerogenes<br>n=7	7	0
**Non fermentative Gram-negative rods**	**Piperacillin tazobactam resistance resistant**	**Carbapenem resistant**
Pseudomonas aeruginosa<br>n=40	9	5
Acinetobacter spp<br>n=5	5	0
**Gram positive cocci**	**Methicillin resistant**	**Ampicillin resistant**
Staphylococcus aureus<br>n=35	5	-
Coagulase-negative *Staphylococcus spp*<br>n=26	0	-
Enterococcus spp<br>n=17	-	0
**Yeasts**	**Azole resistant**	**Echinocandin resistant**
Candida spp<br>n=23	6	0

Table S4. Risk-factors associated with the development of hospital acquired infections according to bivariate analysis

	RR (CI 95%) p
Male sex	**1.63 (1.24 -2.15) p=0.0004**
Age >60 years	1.13 (0.88-1.45) p=0.3361
Obesity	**1.49 (1.16-1.90) p=0.0012**
Diabetes mellitus	1.11 (0.85-1.44) p=0.4385
Hypertension	0.87 (0.67-1.13) p=0.2963
Chronic obstructive pulmonary disease	0.31 (0.05-2.14) p=0.1861
Immunosuppression	1.05 (0.62-1.76) p=0.8610
Cardiovascular disease	1.15 (0.70-1.88) p=0.5903
Chronic kidney disease	1.10 (0.57-2.10) p=0.7836
Liver cirrhosis	0.63 (0.10-4.10) p=0.6154
Charlson's score >2 points	1.02 (0.78-1.32) p=0.8965
Oxygen saturation <90%	2.19 (0.93-5.17) p=0.0547
Lymphocyte count, <800 cells/uL	**1.46 (1.12-1.89) p=0.0039**
C reactive protein ≥10 mg/dL	**2.93 (2.01-4.27) p<0.0001**
Ferritin >500 ng/mL	**2.07 (1.56-2.76) p<0.0001**
Lactic dehydrogenase ≥246 U/L	**2.35 (1.42-3.90) p=0.004**
D Dimer >500 ng/mL	**1.45 (1.14-1.86) p=0.0030**
Use of IMV in the first 24 hours after admission	**8.29 (6.50-10.57) p<0.0001**
Empirical antibiotic-therapy	1.10 (0.85-1.41) p=0.474
Corticosteroid treatment	**2.10 (1.63-2.71) p<0.0001**
Tocilizumab treatment	**1.56 (1.05-2.33) p=0.03542**
Enrollment in a clinical trial	**0.51 (0.34-0.75) p=0.004**

CI confidence interval, *dl* deciliter, *IMV* invasive mechanical ventilation, *IQR* interquartile range, *L* litters, *mg* milligrams, *ml* milliliter, *ng* nanograms, *RR* relative risk, *U* units, *uL* microliter
